# A CMOS Optoelectronic Receiver IC with an On-Chip Avalanche Photodiode for Home-Monitoring LiDAR Sensors

**DOI:** 10.3390/s21134364

**Published:** 2021-06-25

**Authors:** Ji-Eun Joo, Myung-Jae Lee, Sung Min Park

**Affiliations:** 1 Department of Electronic and Electrical Engineering, Ewha Womans University, Seoul 03760, Korea; wxop02@ewhain.net; 2 Graduate Program in Smart Factory, Ewha Womans University, Seoul 03760, Korea; 3 Post-Silicon Semiconductor Institute, Korea Institute of Science and Technology, Seoul 02792, Korea; mj.lee@kist.re.kr

**Keywords:** avalanche photodiode, CMOS, feedforward, limiting amplifier, optoelectronic, transimpedance amplifier

## Abstract

This paper presents an optoelectronic receiver (Rx) IC with an on-chip avalanche photodiode (APD) realized in a 0.18-μm CMOS process for the applications of home-monitoring light detection and ranging (LiDAR) sensors, where the on-chip CMOS P^+^/N-well APD was implemented to avoid the unwanted signal distortion from bondwires and electro-static discharge (ESD) protection diodes. Various circuit techniques are exploited in this work, such as the feedforward transimpedance amplifier for high gain, and a limiting amplifier with negative impedance compensation for wide bandwidth. Measured results demonstrate 93.4-dBΩ transimpedance gain, 790-MHz bandwidth, 12-pA/√Hz noise current spectral density, 6.74-μA_pp_ minimum detectable signal that corresponds to the maximum detection range of 10 m, and 56.5-mW power dissipation from a 1.8-V supply. This optoelectronic Rx IC provides a potential for a low-cost low-power solution in the applications of home-monitoring LiDAR sensors.

## 1. Introduction

Light detection and ranging (LiDAR) sensors have been proliferated for the past decade because they can be applied to diverse fields such as 3-dimensional imaging for unmanned self-driving cars, and medical and industrial applications [1]. Particularly, LiDAR sensors can be a potential solution for home-monitoring elder-care systems because they can inherently provide strong immunity against RF interferences, small form-factor, and blurred images for the sake of portrait right protection [2].

In these LiDAR sensors, avalanche photodiodes (APDs) are mostly exploited as an off-chip optical detector. However, these off-chip APD devices may increase the packaging cost considerably in the cases of multi-channel Rx arrays and deteriorate signal integrity because of the bondwires between APDs and receiver (Rx) chips. Furthermore, on-chip electro-static discharge (ESD) protection diodes may be needed, hence shrinking the Rx bandwidth by the increased input capacitance. Therefore, we present an on-chip CMOS APD in this work which can be a better solution to resolve the aforementioned issues.

Yet, on-chip CMOS APDs suffer from low responsivity and limited bandwidth when compared to the discrete APD devices. Especially, silicon CMOS implementation of on-chip APDs restricts their operations only in the wavelength of 850 nm [3]. Nonetheless, an optoelectronic Rx IC with an integrated on-chip APD is realized in this paper by using a standard 0.18-μm CMOS process. In particular, various circuit techniques are exploited to overcome the inherent defects of on-chip silicon photodiodes.

**Figure 1:**
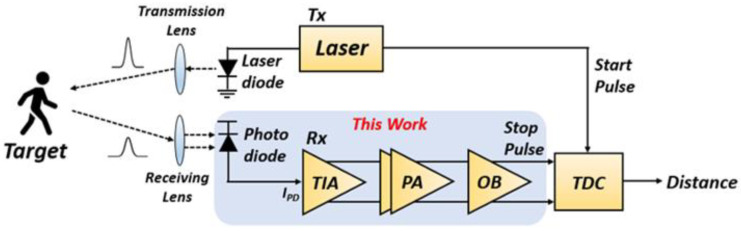
Block diagram of a typical light detection and ranging (LiDAR) sensor.

Figure 1 shows the block diagram of a typical linear-mode LiDAR system, where the Rx consists of a photodiode for light detection, a transimpedance amplifier (TIA) for current-to-voltage signal conversion, a post-amplifier (PA) for voltage-gain boosting, and an output buffer (OB) for 50-Ω impedance matching to a following off-chip circuitry, e.g., time-to-digital converter (TDC). Since an on-chip CMOS APD is integrated with the Rx circuits, this work is named as ‘an optoelectronic Rx IC’.

The target application of this optoelectronic Rx IC is home-monitoring LiDAR sensors for both single elders who live alone and senile dementia patients that reside in either hospitals or nursing homes. Therefore, the optoelectronic LiDAR sensors mandate the following specifications. First, it should be able to detect the distance within 10 m. Second, owing to the lack of responsivity, avalanche multiplication should be equipped in on-chip CMOS photodiodes. Third, the generated photocurrents from on-chip CMOS APDs may be still in the range of a few micro-amperes even with the avalanche multiplication. Therefore, the front-end TIA circuit should provide high transimpedance gain so as to recover the weak incoming signals. In other words, the LiDAR sensors should be able to lower the minimum detectable signal (MDS).

Section 2 describes the realization of an on-chip CMOS APD. Section 3 presents the circuit description of the proposed Rx including a feedforward TIA and a limiting amplifier (LA) with negative impedance compensation. Section IV demonstrates the measured results of the proposed optoelectronic Rx IC realized in a 0.18-µm CMOS process. Lastly, a conclusion follows.

## 2. On-Chip CMOS P^+^/N-Well APD

**Figure 2:**
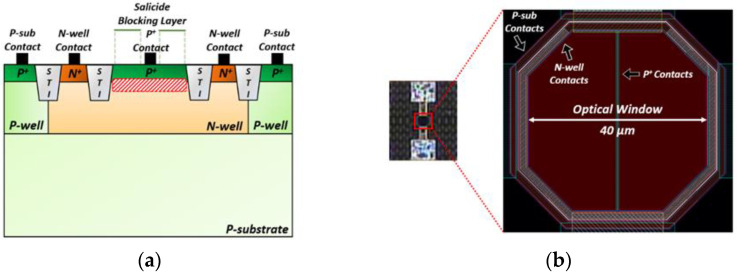
(**a**) Cross-sectional view of the proposed on-chip CMOS P+/N-well avalanche photodiode (APD) and (**b**) its chip photomicrograph and layout.

Figure 2a depicts the cross-sectional view of an on-chip CMOS APD with P^+^/N-well architecture that can yield large avalanche gain owing to the guard ring structure of shallow trench isolation (STI). The avalanche multiplication occurs in the P^+^/N-well junction. It should be noted that the P^+^ contacts in the N-well are connected to the input node of the following TIA circuit, hence excluding the slow diffusion currents contributed from the P-substrate.

The STI guard rings are designed to be deeper in depth than the P^+^/N-well junction, so that the STI can prevent premature edge breakdown. Furthermore, the high electric-field can be distributed uniformly at the planar junction [4].

Figure 2b illustrates the layout of the on-chip CMOS P^+^/N-well APD and its chip microphotograph, where the shape is octagonal to minimize the possible damage from edge breakdown. The P^+^ source/drain region is covered by the salicide blocking layer to form an optical window. However, the P^+^ contacts located in the middle of the optical window cannot be blocked because the salicide process reduces the contact resistivity [5].

Area occupied by the P^+^ contacts should be small enough not to deteriorate the APD responsivity. In this work, the diagonal length of the optical window is designed to be 40 µm.

## 3. Circuit Description

**Figure 3:**
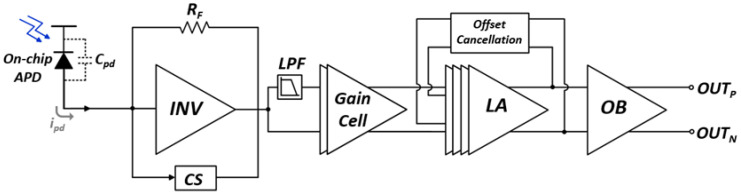
Block diagram of the proposed optoelectronic Rx IC.

Figure 3 depicts the detailed block diagram of the proposed optoelectronic Rx IC which consists of an on-chip P^+^/N-well APD, a feedforward TIA, a three-stage LA with negative impedance compensation, and an OB.

### 3.1. Feedforward TIA

The front-end feedforward TIA determines the overall performance of the optoelectronic Rx IC, therefore mandating stringent requirements of high transimpedance gain to recover weak incoming signals, low noise current spectral density to minimize the MDS, differential signaling for common-mode noise rejection, etc.

**Figure 4:**
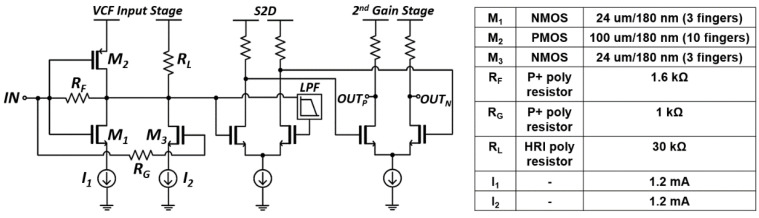
Schematic diagram of the feedforward TIA.

Figure 4 shows the schematic diagram of the feedforward TIA that comprises a conventional voltage-mode inverter (INV) input stage with a feedback resistor (*R_F_*), a feedforward common-source amplifier with its gate connected to the gates of the INV stage, a differential pair with a low-pass-filter for single-to-differential conversion, and an additional differential gain stage for gain boosting further.

According to the small signal analysis of the simplified feedforward input stage (as described in [6]), the transimpedance gain is given by,

(1) $$Z_{T}\left(0\right)=\frac{v_{out}}{i_{in}}=-\frac{\left(g_{m1}+g_{m2}+g_{m3}\right)R_{F}-1}{g_{m1}+g_{m2}+g_{m3}+\frac{1}{r_{o1}\left|\left|r_{o2}\right|\right|r_{o3}||R_{L}}}≅-R_{F}$$

where *g_mi(i=1 to 3)_* and *r_oi(i=1 to 3)_* represent the transconductance and the output resistance of each transistor.

The input-referred noise current spectral density of the simplified feedforward input stage (as described in [6]) is given by,

(2) $${\bar{I^{2}}}_{n,\;TIA}\left(f\right)≅\frac{4kT}{R_{F}}+4kT\Gamma \left(\frac{1}{g_{m1}}+\frac{1}{g_{m2}}\right)\times \left[{\left(2\pi C_{T}\right)}^{2}f^{2}+\frac{1}{R_{F}^{2}}\right]+4kT\left(\Gamma \frac{1}{g_{m3}}+R_{G}\right)\times \;\left[{\left(2\pi C_{T}\right)}^{2}f^{2}+\frac{1}{R_{F}^{2}}\right]≅\frac{4kT}{R_{F}}+4kT\Gamma \left(\frac{1}{g_{m1}}+\frac{1}{g_{m2}}+\frac{1}{g_{m3}}\right)\times {\left(2\pi C_{T}\right)}^{2}f^{2}$$

where *k* is the Boltzmann’s constant, *T* is the absolute temperature, and Γ(≈2) is the Ogawa’s noise factor of a MOSFET. Furthermore, *C_T_ (=C_D_ + C_in,M1_ + C_in,M2_)* represents the total capacitance at the input node of the feedforward TIA which includes the photodiode capacitance (*C_D_*) and the input capacitance of the INV input stage, i.e., *C_in,M1_ + C_in,M2_ = C_gs1_ + C_gs2_ + (1 + A_v_)·(C_gd1_ + C_gd2_)*. It is noted that *R_G_* is set to 1 kΩ in this work so that the noise contribution from R_G_ can be negligible.

Then, the input-referred mean-square noise current is given by,

(3) $${\bar{i^{2}}}_{n,\;TIA}≅\frac{4kT}{R_{F}}BW_{n1}+\frac{4kT\Gamma }{3}C_{T}^{2}BW_{n2}^{2}\left(\frac{1}{g_{m1}}+\frac{1}{g_{m2}}+\frac{1}{g_{m3}}\right)$$

where *BW*_*n*1_ is the noise bandwidth for white noise and *BW*_*n*2_ is the noise bandwidth for *f*^2^ noise. For *Q* = 1/$\sqrt{2}$, *BW_n*1*_* ≈ 1.11·*BW^3^_3dB_* and *BW_n*2*_* ≈ 1.49·*BW_3dB_* [7].

In Figure 4, the value of *R_L_* is selected to a few tens of kilo-ohm. Then, the bias current (I_2_) of M_3_ can be mostly supplied through the PMOS (M_2_) of the INV stage, because the DC drain voltage of M_2_ is fixed by the action of the INV stage via the feedback resistor (*R_F_*). Hence, this action enables to boost the transconductance (*g_m2_*) of M_2_ and helps to reduce the noise current spectral density of the feedforward TIA.

**Figure 5:**
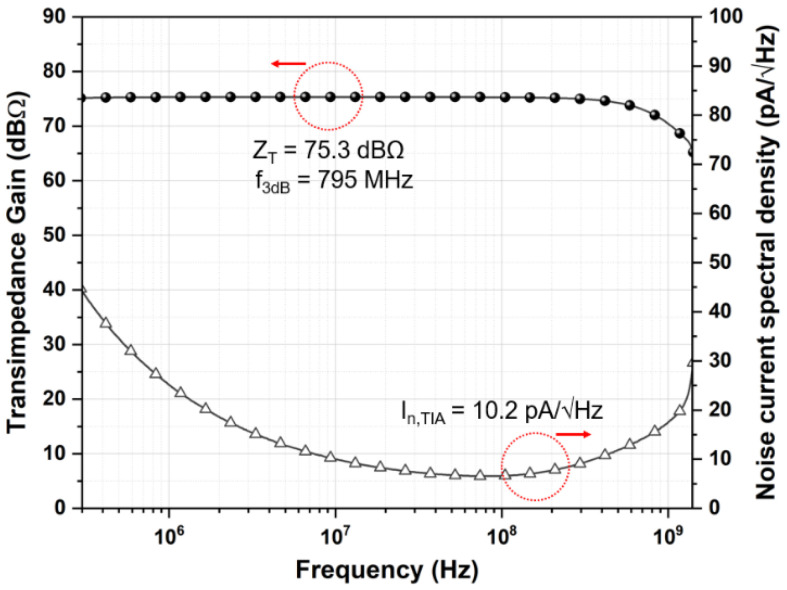
Simulated frequency response of the feedforward TIA.

Post-layout simulations were conducted by utilizing the model parameters of a standard 0.18-µm CMOS process. Figure 5 shows the simulated frequency response where the feedforward TIA achieves the differential transimpedance gain of 75.3 dBΩ, the bandwidth of 795 MHz, and the average input-referred noise current spectral density of 10.2 pA/√Hz which leads to the input referred root-mean-square (RMS) noise current of 287 nA_rms_.

### 3.2. Limiting Amplifier

**Figure 6:**
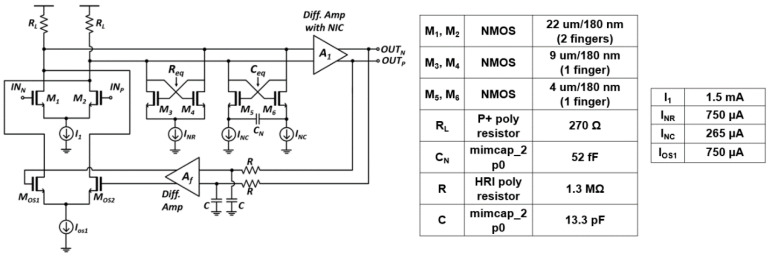
Schematic diagram of the LA with negative impedance compensation.

Limiting amplifier (LA) amplifies the small output voltage signals of the feedforward TIA to sufficient levels so that a following TDC can detect the signals precisely. Furthermore, the bandwidth of LA should be wide enough not to degrade the operating speed of the preceding TIA. Figure 6 depicts the block diagram of the proposed LA which consists of three cascaded gain cells and an offset cancellation network. In each gain cell, the negative impedance compensation techniques are incorporated to ensure wide bandwidth and high voltage gain [8] because a substantial voltage gain cannot be obtained in a conventional differential amplifier owing to its unavoidable voltage headroom issue.

The schematic diagram of a gain-cell is shown in Figure 6, where the active negative resistance circuit can alleviate the voltage-headroom issue. However, a rather large output resistance and the unavoidable parasitic capacitance occurring from the active devices may limit the bandwidth significantly. Therefore, a negative capacitance circuit is added in parallel so that the bandwidth can be extended by canceling the output capacitance.

Small signal analysis shows that the equivalent resistance and capacitance are given by,

(4) $$R_{eq}=-\frac{2}{g_{m}}$$

(5) $$C_{eq}=-\frac{1}{sC_{N}}\times \left(\frac{g_{m}+s\left(C_{gs}+2C_{N}\right)}{g_{m}}\right)$$

where *g_m_* represents the transconductance of transistors in the negative impedance circuits and *C_gs_* represents the gate-source capacitance of transistors in the negative capacitance circuit.

Moreover, a DC offset network should be employed because an input offset voltage, however tiny it may be, can be amplified to saturate the output of the LA. Figure 6 shows the proposed offset cancellation feedback network, where an RC low-pass filter is utilized to extract the DC components from the output signals. Then, the extracted DC offset voltage is buffered by an error amplifier with a voltage gain of *A_f_*, consequently removing the offset voltage.

Even though the input offset voltage is suppressed by *A_f_*, a low cutoff-frequency is newly introduced at $\frac{1}{2\pi }\times \frac{A_{v,LA}A_{f}+1}{RC}$ where *A_v,LA_* is the voltage gain of the LA [9]. Therefore, A_f_ should be judiciously selected to a reasonable value so that the feedback network maintains the circuit stability.

**Figure 7:**
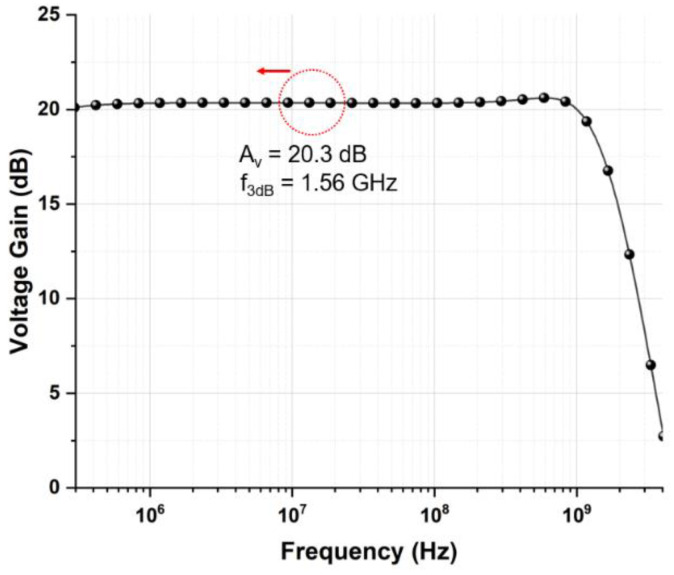
Simulated frequency response of the proposed limiting amplifier (LA).

Figure 7 depicts the simulated frequency response of the proposed LA which reveals the voltage gain of 20.3 dB and the bandwidth of 1.56 GHz.

## 4. Measured Results

**Figure 8:**
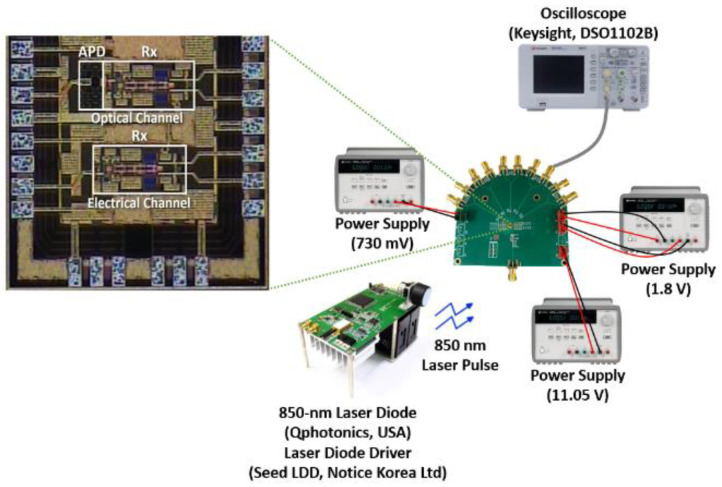
Chip microphotograph of the proposed optoelectronic Rx IC and its test setup.

Test chips of the proposed optoelectronic Rx IC were fabricated in a standard 0.18-μm CMOS process. Figure 8 shows the chip microphotograph and its test setup, in which the chip core occupies an area of 522 × 171 μm^2^. DC measurements reveal that the optoelectronic Rx IC consumes 56.5 mW from a single 1.8-V supply.

**Figure 9:**
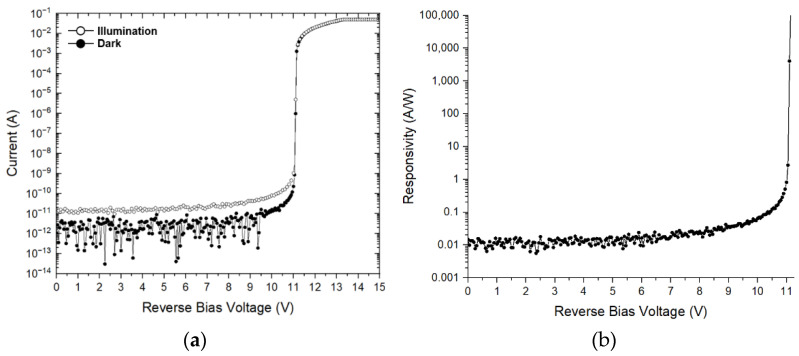
(**a**) Measured I–V curve of the P^+^/N-well APD and (**b**) its responsivity.

Figure 9a demonstrates the measured current-voltage characteristics of the on-chip CMOS P^+^/N-well APD under the conditions of both dark and optical illuminations. It is clearly seen that the dark current and the illumination current rise sharply at the breakdown voltage of 11.1 V owing to the avalanche multiplication process, where the incident optical power is about –60 dBm. The responsivity (R) of the on-chip CMOS P^+^/N-well APD is then given by,

(6) $$R=\frac{I_{illumination}-I_{dark}}{P_{opt}}$$

where *I_illumination_* and *I_dark_* represent the measured currents under illumination and dark conditions, respectively. P_opt_ represents the incident optical power. Figure 9b shows the measured responsivity versus bias voltages, where the responsivity of 2.72 A/W is acquired at the reverse bias voltage of 11.05 V.

**Figure 10:**
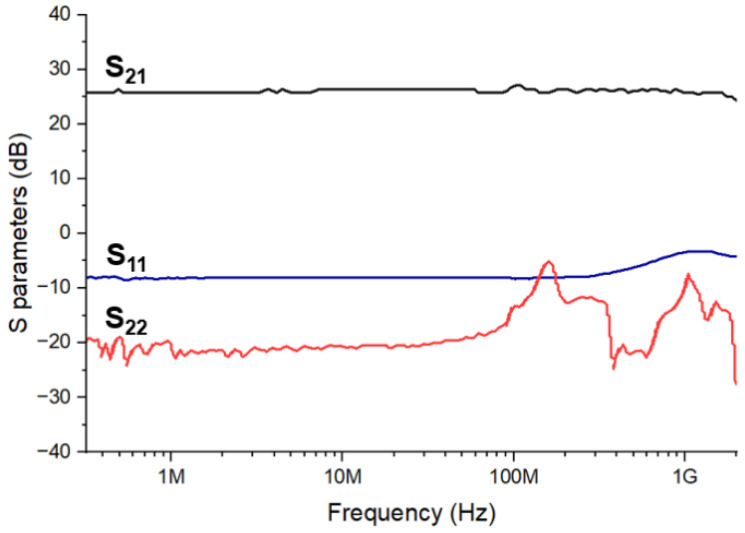
Measured S-parameters of the proposed Rx IC.

Figure 10 demonstrates the measured S-parameters of the optoelectronic Rx IC, where S_21_ of 26 dB are achieved with a 50-Ω termination. It leads to the single-ended transimpedance gain (Z_21_) of 87.4 dBΩ by the following equation,

(7) $$Z_{T}=\frac{2S_{21}Z_{0}}{\left(1-S_{11}\right)\left(1-S_{22}\right)-S_{21}S_{12}}$$

**Figure 11:**
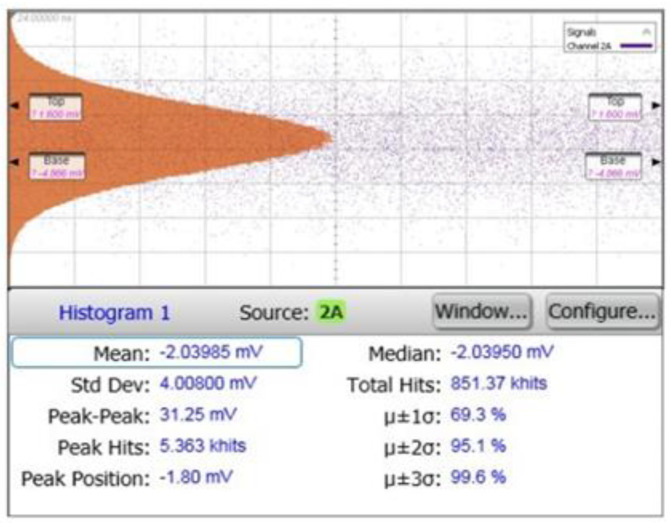
Measured noise output voltage of the proposed Rx IC.

Figure 11 shows the measured output noise voltage of the optoelectronic Rx IC. Considering the background noise of the utilized oscilloscope (Agilent DCA 86100D), the input referred average noise current spectral density is given by [10],

(8) $$I_{n,in}\equiv \frac{2\sqrt{{\left(4.01\;\mathrm{mV}\right)}^{2}-{\left(0.660\;\mathrm{mV}\right)}^{2}}}{87.4\;\mathrm{dB}\Omega }=337\;nA_{rms}$$

(9) $$I_{n,in,avg}\equiv \frac{I_{n,in}}{\sqrt{790\;\mathrm{MHz}}}=12\;\mathrm{pA}/\sqrt{\mathrm{Hz}}$$

This measured input-referred RMS noise of 337-nA_rms_ is then translated to 6.74-µA_pp_ MDS since the signal-to-noise ratio (SNR) of 10 is required for successful detection [11]. With APD responsivity of 2.72 A/W, it corresponds to the minimum incident optical power of 2.48 µW. Assuming that 1-mW laser power is emitted, the detection range can be estimated to be 10 m. Therefore, the measured noise performance of the optoelectronic Rx IC satisfies the design specification for indoor home-monitoring LiDAR sensors.

**Figure 12:**
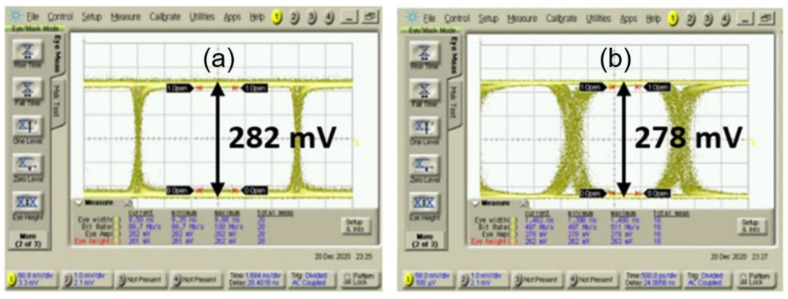
Measured eye-diagrams at (**a**) 100 Mb/s, (**b**) 500 Mb/s for 50 µA_pp_ 2^31^-1 PRBS input.

Figure 12 shows the measured eye-diagrams for 50-μA_pp_ 2^31^-1 pseudo random bit sequence (PRBS) inputs at different operation speeds of 100 Mb/s and 500 Mb/s, respectively. Measurements confirm that the proposed optoelectronic Rx IC achieves wide and clean eyes up to 800-Mb/s operations.

**Figure 13:**
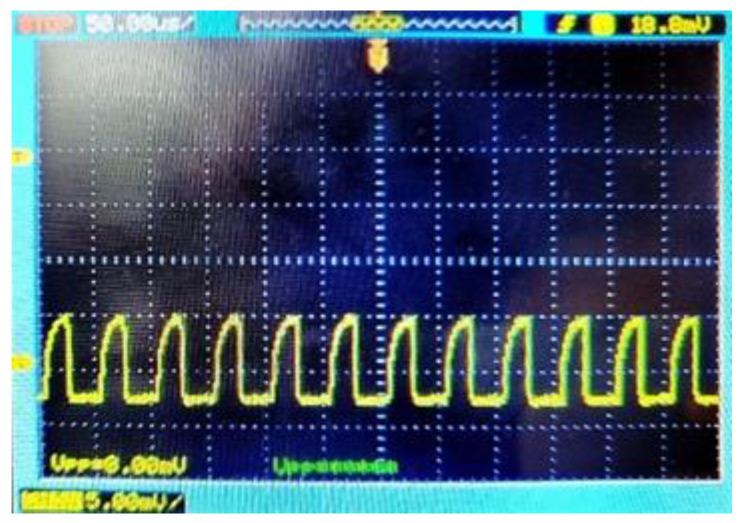
Optically measured pulse response of the proposed optoelectronic Rx IC.

Figure 13 demonstrates the optically measured pulse responses, where the light pulses were generated by utilizing an 850-nm laser source driver (Seed LDD, Notice Korea Ltd.) with a laser diode (Qphotonics, USA). The consecutive light pulses were incident on the on-chip CMOS P^+^/N-well APD, clearly showing the final output voltage pulses.

Table 1 summarizes and compares the performance of the proposed optoelectronic Rx IC with other prior arts. In [6], a 16-channel off-chip InGaAs PIN-PD array module with 0.9-A/W responsivity was utilized. Therefore, it could not avoid the increase of cost and form factor. References [12,13,14,15] exploited off-chip APDs, which resulted in hardware complexity in an array configuration of multi-channel receivers.

This work is the first attempt to integrate an on-chip CMOS APD with analog frontend IC for the applications of LiDAR sensors. Despite the well-known inferior performance of an on-chip CMOS APD, the proposed optoelectronic Rx IC provides comparable performance with a little expense of noise degradation. Yet, it would barely be a critical issue for home-monitoring sensors with 10-m detection range.

## 5. Conclusions

The first CMOS optoelectronic Rx IC was realized for the applications of indoor home-monitoring LiDAR sensors. The on-chip P^+^/N-well APD demonstrates the responsivity of 2.72 A/W at the reverse bias voltage of 11.05 V, and the whole optoelectronic Rx IC achieves the differential transimpedance gain of 93.4 dBΩ, bandwidth of 790 MHz, and maximum detection range of 10 m with power consumption of 56.5 mW. It can be concluded that this work certainly provides a low-cost solution for short-range indoor LiDAR sensors.

## Figures and Tables

**Table 1:** Performance summary and comparison of the proposed optoelectronic Rx IC with other prior arts.

Parameters		This Work	[6]	[12]	[13]	[14]	[15]
Technology (nm)		CMOS 180	CMOS 180	HV-CMOS 180	CMOS 130	CMOS 180	CMOS 350
PD	Type	APD (on-chip)	InGaAs PIN-PD (off-chip)	APD (off-chip)	APD (off-chip)	APD (off-chip)	APD (off-chip)
	C_pd_ (pF)	0.5 *	0.5	0.5	2	1.2	1.2
	Responsivity (A/W)	2.72	0.9	N/A	N/A	50	N/A
	Wavelength (nm)	850	1550	N/A	N/A	905	N/A
TZ gain (dBΩ)		93.4	76.3	88	78	86	100
Bandwidth (MHz)		790	720	700	640	281	450
Noise current spectral density (pA/√Hz)		12	6.3	17	4.7	4.68	2.59
Power Dissipation (mW)		56.5	29.8	180	114	200	6.6

* estimated from the measured breakdown voltage.

## Data Availability

Not applicable.
